# FloCyT: A Flow-Aware Centroid Tracker for Cell Analysis in High-Speed Capillary-Driven Microfluidic Flow

**DOI:** 10.3390/s25227040

**Published:** 2025-11-18

**Authors:** Suraj K. Maurya, Matt Stark, Cédric Bessire

**Affiliations:** 1 Bio/CMOS Interfaces Laboratory, Ecole Polytechnique Federale de Lausanne (EPFL), Rue de la Maladiere 71, 2000 Neuchatel, Switzerland; 2 Institute For Human Centered Engineering, Berner Fachhochschule (BFH), Quellgasse 21, 2501 Biel, Switzerland

**Keywords:** image flow cytometry, capillary-driven flow, microfluidic cell tracking, centroid-based tracking, anisotropic gating, multi-object tracking, point-of-care diagnostics, cytological analysis

## Abstract

Capillary-driven microfluidic chips have emerged as promising platforms for point-of-care diagnostics, offering portable, inexpensive, and pump-free operation. Accurate tracking of cell flow in these systems is vital for quantitative applications such as on-chip cytometry, cell counting, and biomechanical analysis. However, tracking in capillary-driven devices is challenging due to rapid cell displacements, flow instabilities, and visually similar cells. Under these conditions, conventional tracking algorithms such as TrackPy, TrackMate, SORT, and DeepSORT exhibit frequent identity switches and trajectory fragmentation. Here, we introduce FloCyT, a robust, high-speed centroid tracking tool specifically designed for capillary-driven and microfluidic flow. FloCyT leverages microchannel geometry for tracking and uses anisotropic gating for association, global flow-aware track initialisation, and channel-specific association. This enables precise tracking even under challenging conditions of capillary-driven flow. FloCyT was evaluated on 12 simulated and 4 real patient datasets using standard multi-object tracking metrics, including IDF1 and MOTA, ID switches, and the percentage of mostly tracked objects. The results demonstrate that FloCyT outperforms both standard and flow-aware-modified versions of TrackPy and SORT, achieving higher accuracy, more complete trajectories, and fewer identity switches. By enabling accurate and automated cell tracking in capillary-driven microfluidic devices, FloCyT enhances the quantitative sensing capability of image-based microfluidic diagnostics, supporting novel, low-cost, and portable cytometry applications.

## 1. Introduction

Microfluidics has proven itself as a promising technology for point-of-care (POC) applications due to its ability to provide a functional lab-on-a-chip with minimal equipment requirements. These chips offer solutions not only for analyzing biological samples but also for preparing them through enrichment, reactions, and even on-chip imaging [1]. In particular, capillary-driven microfluidic chips, which autonomously drive fluid through microchannels via capillary action, have gained attention for portable diagnostics [1,2]. They are compact and can be easily manufactured from inexpensive polymer materials, e.g., polymethyl methacrylate (PMMA) or others, rendering them particularly suitable for single-use diagnostic assays. Many diagnostic workflows involve imaging the sample, followed by image processing or machine learning-based approaches for cell detection and tracking to extract quantitative biological parameters [3,4,5,6,7]. Accurate tracking of cells flowing through such chips is crucial for applications like on-chip flow cytometry, cell counting, and biomechanical assays, as it allows real-time quantification much like a bench-top flow cytometers.

Unlike pump-driven microfluidic systems, where cells typically move at a uniform speed, capillary-driven systems pose greater challenges for tracking because of flow instabilities, rapid cell displacements (often spanning hundreds of pixels per frame), and the complex dynamics exhibited by cells under varying flow velocities. Conventional tracking tools such as TrackPy [8] and TrackMate [9] assume small displacements between frames, relying on isotropic search windows or linear assignment problem (LAP) frameworks [10], which break down under these conditions. Multi-object tracking frameworks such as SORT and DeepSORT [11,12] incorporate Kalman prediction and data association. Their reliance on slow-motion assumptions or appearance makes them unsuitable for capillary-driven microfluidic cell tracking, where cells are visually similar and move rapidly. As a result, generic tracking algorithms are prone to track fragmentation and identity switches in high-speed capillary-driven microfluidic assays.

To overcome these limitations, we present FloCyT, a dedicated tracker that leverages microchannel geometry and flow direction to improve robustness. FloCyT diverges from conventional centroid trackers through three core advancements: incorporation of prior global flow knowledge, anisotropic search gating aligned with the flow axis, and channel-specific data association to maintain identity across parallel lanes. Together, these enable reliable, high-speed tracking even where traditional methods fail. We benchmark FloCyT against both TrackPy and SORT, in their original forms and modified global flow-aware variants, using 12 simulated datasets and 4 real patient datasets, using Multi-Object tracking metrics (MOT) designed for the Classification of Events, Activities, and Relationships (CLEAR) evaluation called the CLEAR-MOT metrics framework to measure tracking accuracy [13].

## 2. Methods

FloCyT’s framework is based on the Kalman filter and Hungarian assignment paradigm, similar to the SORT tracker [11]. However, unlike SORT, which propagates bounding boxes and relies on intersection-over-union (IoU) for association, FloCyT performs centroid-based tracking with an anisotropic gated cost function aligned to the microfluidic flow axis. In addition, FloCyT supports channel-specific data association and user-defined region-of-interest (ROI) track lifecycle management, ensuring consistent tracking in capillary-driven flow.

### 2.1. State Estimation

FloCyT models each cell movement with a linear Kalman filter whose state vector contains the two-dimensional centroid position and velocity given by Equation (1).

(1) $$x={\left[\;d^{x},\;d^{y},\;v_{0}^{x}\left(t\right),\;v_{0}^{y}\left(t\right)\;\right]}^{T}$$

where $d^{x}$ and $d^{y}$ are the detected centroid coordinates and $v_{0}^{x}\left(t\right)$ and $v_{0}^{y}\left(t\right)$ are the initial velocities obtained by Equation (5). This formulation assumes approximately constant velocity between consecutive frames, with process noise capturing local fluctuations caused by flow instabilities or cell–cell interactions. At each time step, the filter performs a prediction to estimate the next centroid location, followed by a correction whenever a detection is successfully associated.

### 2.2. Anisotropic Gated Cost Matrix and Data Association

To associate predicted cell positions with detections, we define a flow-aware cost function. Let $p_{i}=\left(p_{i}^{x},p_{i}^{y}\right)$ denote the *i*-th predicted position for $i=1,\ldots ,N_{p}$, where $N_{p}$ is the number of predicted tracks, and $d_{j}=\left(d_{j}^{x},d_{j}^{y}\right)$ the *j*-th detected position for $j=1,\ldots ,N_{d}$, where $N_{d}$ is the number of detections in the current frame.

The displacements between prediction *i* and detection *j* are defined in Equation (2).

(2) $$\Delta_{\|}^{ij}=d_{j}^{x}-p_{i}^{x},\;\Delta_{⊥}^{ij}=d_{j}^{y}-p_{i}^{y},$$

where $\Delta_{\|}^{ij}$ is the downstream or upstream displacement and $\Delta_{⊥}^{ij}$ is the orthogonal displacement.

We introduce a binary mask $M_{ij}$ to enforce the gating condition given by Equation (3).

(3) $$M_{ij}=\left\{\begin{array}{cc} 1, & -s_{\mathrm{bk}}\leq \Delta_{\|}^{ij}\leq s_{\mathrm{fr}}\;\;\mathrm{and}\;\;\left|\Delta_{⊥}^{ij}\right|\leq s_{\mathrm{ort}},\\ 0, & \mathrm{otherwise}, \end{array}\right.$$

where $s_{\mathrm{fr}}$, $s_{\mathrm{bk}}$, and $s_{\mathrm{ort}}$ are the forward, backwards, and orthogonal gating thresholds, respectively. They can be user-defined parameters or be calculated via the time-dependent displacement from the current velocity estimate $v_{0}\left(t\right)$.

The assignment cost between prediction *i* and detection *j* is given by Equation (4).

(4) $$D_{ij}=\left\{\begin{array}{cc} {∥d_{j}-p_{i}∥}_{2}, & M_{ij}=1,\\ +\infty , & M_{ij}=0, \end{array}\right.$$

where $∥d_{j}-p_{i}{∥}_{2}$ is the Euclidean distance between the predicted and detected positions.

$D_{ij}$ restricts associations to an anisotropic search region, which we have chosen to elongate along the flow direction and narrow in the orthogonal direction, reflecting the physical reality of capillary-driven flows in microfluidic channels. Finally, the cost matrix $D\in R^{N_{p}\;\times \;N_{d}}$ is used in the linear assignment (Hungarian) algorithm to find the best matches, considering only feasible pairs where $D_{ij}<\infty$.

### 2.3. Track Creation and Deletion

*Track Creation:*

A new Kalman-based tracker is created for each unmatched detection whose centroids lies within the ROI. The initial velocity is estimated as a weighted average of a global velocity estimate $v_{g}\left(t\right)$ and a local velocity estimate $v_{l}\left(t\right)$ given by Equation (5).

(5) $$v_{0}\left(t\right)=\alpha \;v_{l}\left(t\right)+\left(1-\alpha \right)\;v_{g}\left(t\right),\;0\leq \alpha \leq 1.$$

where, $v_{l}\left(t\right)$ is the component-wise median of recent track velocities from the Kalman filter, and $v_{g}\left(t\right)$ is obtained using the method described in Section 2.4. At the beginning of the analysis, α is set to zero so that $v_{0}\left(t\right)$ relies only on the global velocity estimate. Once a sufficient number of active tracks are available, α is increased (e.g., to 0.7), giving greater weight to the local median velocity. This staged initialization allows the tracker to quickly adapt to the high velocities and temporal variations observed in capillary-driven flows, which would otherwise be difficult to capture reliably.

*Track Deletion:*

An active track is deleted when it no longer provides reliable information. Specifically, each track maintains a counter of consecutive missed frames. If a track remains unmatched for more than the user-defined number of frames, it is terminated. In addition, a track is removed once its estimated position leaves the ROI along the flow axis, ensuring that only cells present within the observable channel segment are retained. These rules prevent stale tracks from persisting after a cell exits the ROI or becomes occluded for too long, thereby maintaining a clean and reliable set of active trajectories.

### 2.4. Tracking Initialization

Objects entrained in laminar channel flow usually have a dominant flow direction and flow speed. An estimate of this dominant motion, the global velocity estimate $v_{g}\left(t\right)$, is used for initial velocity estimation when tracking individual particles in Equation (5).

Let $d=[d^{x},d^{y}]$ denote the centroid coordinates of an individual object, and let $a={\left[d_{1,1},d_{1,2},...,d_{1,m}\right]}^{T}$ denote the centroids of all *m* objects detected in the first frame. Similarly, let $b={\left[d_{2,1},d_{2,2},...,d_{2,n}\right]}^{T}$ denote the centroids of all *n* objects detected in the second frame.

We propose a maximum likelihood estimator of the global velocity from two successive frames using the following steps:

1. Calculate the displacement between all centroids in the first frame and all centroids in the second frame according to Equation (6).
  (6) $$\Delta^{m,n}=b_{n}-a_{m}$$
2. Create a 2D histogram of all displacement vectors in Δ to estimate the motion probability density.
3. Smooth the histogram with a 2D Gaussian kernel with an appropriate size. The kernel size should chosen so displacement patterns arising from the bulk motion are emphasized. It should be large enough to provide robust estimation, but not so large that it over-smooths motion patterns.
4. Select the histogram bin with the highest smoothed density value as the as maximum likelihood estimate of the global velocity vector.

A key advantage of this approach is its minimal user-defined parameter requirements. The only user-defined parameter is the size of the Gaussian kernel used for histogram smoothing; no explicit displacement thresholds or directional priors are required. Larger Gaussian kernels yield smoother probability surfaces and improve robustness when fewer displacement vectors are available, whereas smaller kernels preserve finer detail when a large number of displacement vectors are available.

Bulk velocity estimation can be made more robust by collecting velocity vectors from consecutive frame pairs before calculating the histogram and smoothing. This temporal collection reduces the effect of local fluctuations and should be limited when there is high flow variability, as long-term collection may mask transient flow changes.

Applying kernel density estimation to the histogram instead of each velocity vector has computational advantages. The computation time is bounded by the histogram size instead of the number of displacement vectors in Δ which can be very large for dense flows over many frames.

## 3. Real Dataset Preparation

**Figure 1:**
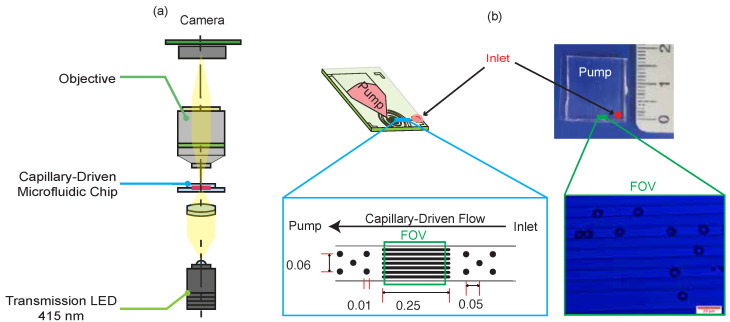
(**a**) Optical setup and placement of the capillary-driven microfluidic chip. (**b**) Typical chip design (left) and the final fabricated chip shown alongside a ruler for comparison (in cm). The blue box highlights the inlet channel structures and their typical dimensions (in mm). The green box indicates the FOV, where the dark discoid structures correspond to RBCs. The red scale bar represents 20 µm.

Blood samples from four patients were provided by the University Hospital Bern (Inselspital). Each sample was diluted with Phosphate-buffered saline (PBS) (pH 7.2; Sigma-Aldrich, St. Louis, MO, USA, Product No. 806544) at a ratio of 1:20. Then, 5 µL of the diluted sample was placed at the inlet of the capillary-driven chip, enabling the cells to flow passively through the microchannels. Video recordings of the flowing cells were captured using a microscope with a transmission light wavelength of 415 nm at a frame rate of 30 fps, as shown in Figure 1a. A typical capillary-driven chip design and and the final chip fabricated using polydimethylsiloxane–polyethylene oxide (PDMS–PEO) are shown in Figure 1b. During fabrication, the PDMS–PEO mixture was prepared by mixing the elastomer base and curing agent in a 10:1 ratio (Sylgard 184, PDMS kit), with 0.1–0.2% (*w*/*w*) dimethylsiloxane (60–70% ethylene oxide) block copolymer (Gelest, Morrisville, PA, USA, Product number: DBE-712) to make the PDMS hydrophilic [14]. In Figure 1b, the green rectangle highlights the parallel grid structures within the field of view (FOV) and the blue box indicates the inlet channel structures. The circular pillars before and after the FOV act as spacer filters to break up agglomerations and prevent channel collapse onto the glass surface. The grid structures in the FOV restrict the lateral motion of red blood cells (RBCs).

In image flow cytometry, the channel width is typically designed to be comparable to the cell diameter to enable single-cell analysis [7]. This configuration maximizes throughput by accommodating multiple parallel channels on a single FOV. Moreover, matching the channel width to the cell diameter improves both detection and tracking accuracy. The geometric confinement limits lateral displacement, ensuring that cells predominantly move along the flow direction. This unidirectional motion reduces trajectory overlap and occlusion, simplifies detection, and enhances the robustness of tracking algorithms.

Unlike externally pumped systems, in capillary pump-driven devices, the flow rate is not constant for a given liquid due the change in pressure gradient over time. It varies with the pump geometry and the filling state of the pump, which makes tracking more challenging. The time-dependent behavior of capillary-driven flow, including pump design optimization, filling dynamics, and CFD modelling of such systems, is discussed in detail by Tavakolidakhrabadi et al. [15].

In our study, the microfluidic pump structures were fixed and channel depth was kept at 5 µm to prevent flipping of RBCs. However, we varied the parallel channels’ width and the number of channels between samples to generate complex flow scenarios by allowing limited lateral movements or to accommodate the magnification from microscope objective as well as to improve the throughput (details in Appendix A).

**Remark** **1.** *In our setup, one pixel corresponds to 0.1548 μm and all the datasets in this study is captured at 30 frames per second (fps). Cell displacement in the video is observed in pixel units. The displacement of cells per frame depends primarily on two factors: (i) the actual physical flow rate in the microfluidic channel and (ii) the frame rate at which the video is captured. For instance, if cells move at an average velocity of 1 mm/s, a video recorded at 30 fps would show a displacement of approximately 200 pixels per frame, whereas recording the same flow at 60 fps would show about 100 pixels per frame. Therefore, from this point onward, we report motion in pixels or pixels per frame rather than in mm/s to avoid ambiguity.*

**Figure 2:**
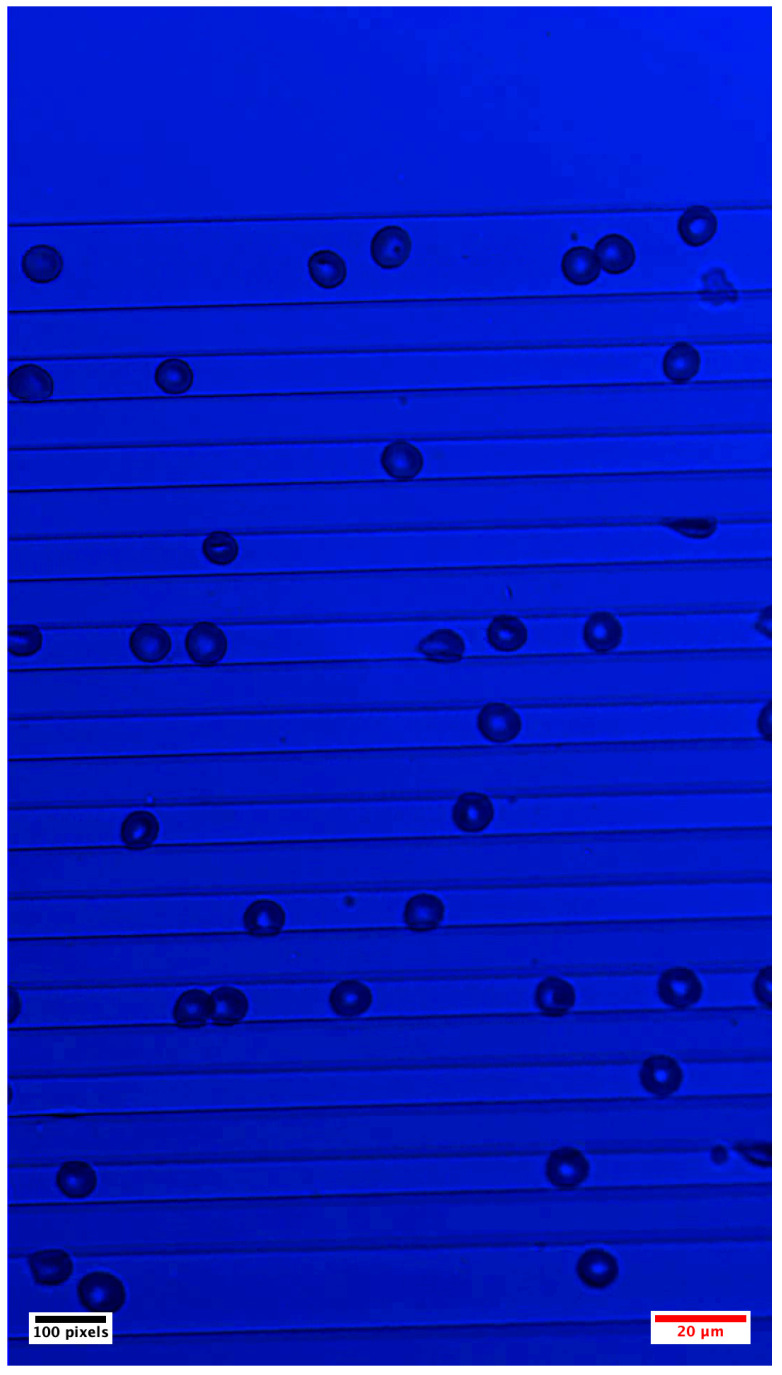
Representative frame showing human blood cells flowing in a microfluidic channel under capillary-driven flow. The dark discoid structures correspond to RBCs. The cells flow from left to right. Channel width and magnification may vary depending on the application requirements. The black scale bar corresponds to 100 pixels, and the red scale bar corresponds to 20 µm. Camera resolution is 1080 × 1920 pixels.

Figure 2 shows a representative frame of patient blood cells flowing in the microfluidic channel. The acquired data was manually annotated and the data characteristics are summarized in Table 1. Additional details such as channel orientation, magnification and number of the channel is presented in the Appendix B, Figure A2.

## 4. Synthetic Dataset Generation

**Figure 3:**
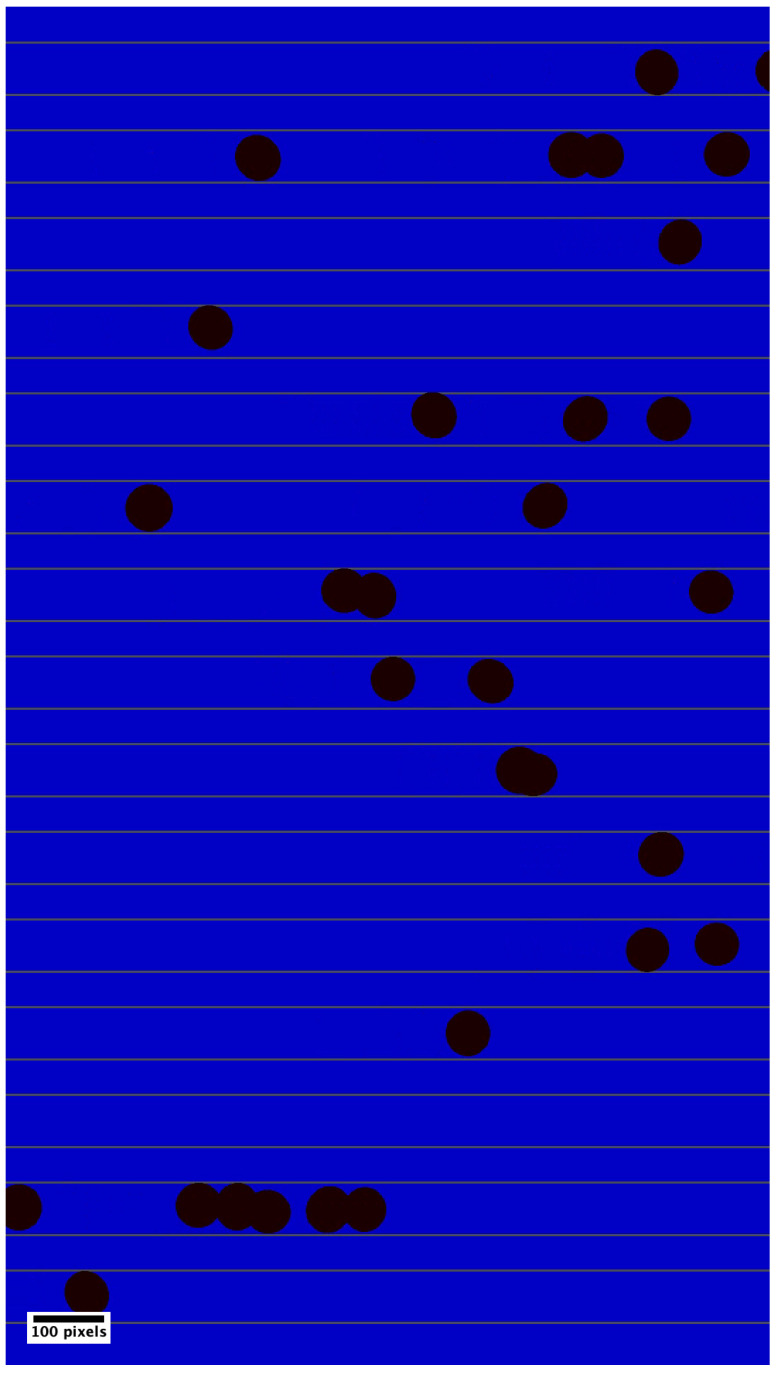
Representative frame of synthetic dataset: dark objects represent cells and horizontal lines represent channel walls. Object movement takes place from left to right. Scale bar is 100 pixels.

To enable more controlled testing of the tracking algorithms beyond the real dataset, we generate synthetic datasets for stress testing of the algorithms. Our simulation does not aim to reproduce the complex biomechanics of RBCs. Instead, we use simplified elliptical particles with controlled motion, heterogeneity, and noise as shown in Figure 3. This design is consistent with established tracking evaluation practice, where synthetic data serves to create known ground truth and systematically vary in difficulty [10,16]. This synthetic stress testing complements the real capillary-driven datasets and enables systematic evaluation of the robustness of the algorithm under high particle density, occlusions, and heterogeneous flow conditions. For synthetic data generation, we used the Python (version 3.9.6) open-source scientific library NumPy (version 2.2.5) to model the movement of ellipse centroids and OpenCV (version 4.11.0) to generate images and videos. The centroid positions, frame numbers, average ellipse radii, and ellipse IDs were stored in a CSV file, which served as ground truth for stress-testing the tracking algorithms.

Each cell *i* is initialized with a constant base velocity $v_{0,i}$, randomly chosen from a normal distribution in interval $\left[v_{\min},\;v_{\max}\right]$ to emulate heterogeneity of the flow among the cells. A global modulation term applied to all cells emulates temporal variation in the flow, as given by Equation (7) where *t* is the frame index and *N* is the total number of frames. A subset of cells is randomly selected to emulate stopping and accelerating behavior during the flow, and their states are maintained separately.

(7) $$m\left(t\right)=1+0.15\cos\;\left(\frac{2\pi t}{N}\right)$$

The effective per frame displacements of the *i*-th cell are defined as follows:

$$\begin{array}{cc} \Delta x_{i}\left(t\right) & =m\left(t\right)\;v_{x,i}\left(t\right)+\eta_{x}\left(t\right),\\ \Delta y_{i}\left(t\right) & =v_{y,i}\left(t\right)+\eta_{y}\left(t\right), \end{array}$$

where

- $v_{x,i}\left(t\right)$ is the longitudinal velocity of cell *i*, which may be updated by the stop/deceleration logic;
- $v_{y,i}\left(t\right)$ is the lateral velocity of cell *i* within the channel;
- $\Delta x_{i}\left(t\right),\Delta y_{i}\left(t\right)$ are the per-frame displacements in the longitudinal and lateral directions, respectively;
- $\eta_{x}\left(t\right),\eta_{y}\left(t\right)$ are Gaussian noise terms modeling uncertainty in the centroid position;
- $v_{\min},v_{\max}$ denote the global bounds for the base velocity distribution.

**Figure 4:**
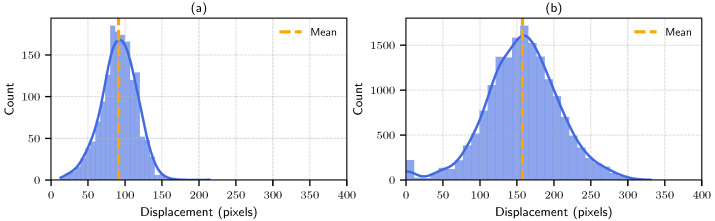
Displacement distributions of cells between consecutive frames for one dataset. (**a**) shows the real dataset of RBC flow velocity variation; (**b**) shows the simulated dataset with higher velocities and greater speed variations.

For the simulated datasets we fixed the channel width to be approximately equal to the cell diameter for 15 parallel channels (see Figure 3), similar to real datasets 1 and 4 (refer to Appendix A). Twelve datasets were simulated to capture low, medium, and high heterogeneity in cell flow. For each condition, different throughputs (cells/s) flowing through the field of view was considered to vary the complexity. All simulations were run for 1500 (at 30 fps) frames with a frame size of 1080 × 1920, using zero-mean Gaussian noise terms $\eta_{x}\left(t\right)$ and $\eta_{y}\left(t\right)$ with standard deviations 5 and 0.5 respectively. The details of the synthetic datasets are summarized in Table 2. The smaller the $v_{min}$, the more the possibility for the high-speed cells to overtake, hence more overlapping, resulting in more complexity for the tracking algorithms. The higher the $v_{min}$ the cells will be visible in the field of view for only a few consecutive frames; therefore, more cells can be added to increase the throughput. Figure 4 provides a comparison between the displacement distribution profile of the cells between consecutive frames. Panel (a) shows the displacement distribution of the real dataset 4; panel (b) shows the displacement distribution of the simulated dataset L.

### Benchmarking

The real datasets and simulated datasets, summarized in Table 1 and Table 2, were used to evaluate and compare six tracking algorithms: FloCyT, FloCyT per channel, TrackPy-original, TrackPy-modified, SORT, and SORT-modified. FloCyT was run with parameters $s_{\mathrm{fr}}=600$, $s_{\mathrm{bk}}=200$, and $s_{\mathrm{ort}}=100$. In the FloCyT per channel variant, KMeans clustering was applied to cluster the centroids in the channel-wise fashion then tracking was done on each cluster with the same search parameter as FloCyT. This variant minimizes the cross-channel association. TrackPy-modified incorporated the initialization algorithm described in Section 2.4 into the TrackPy framework, while the baseline TrackPy was used in its standard form; both were run with a search range of x and y [600,100] respectively. The baseline SORT algorithm was applied with its standard parameters, and SORT-modified extended it by integrating the initialization strategy described in Section 2.4. For FloCyT, FloCyT per channel, TrackPy-modified, and SORT-modified used 15 consecutive frames and sigma=10 was used to estimate global velocity $v_{g}\left(t\right)$. The common parameters were kept the same for all the tracking algorithms for better comparability.

Performance was assessed using the CLEAR MOT metrics framework [13] using open-source Python implementation py-motmetrics [17]. Since the requirements for cell detection vary depending on the application, we did not employ an automatic detection method. Instead, ground truth centroids were used as detections, which eliminates classical detection errors (misses and false positives). Consequently, the following metrics are the most informative for performance comparison.

1. Multiple Object Tracking Accuracy (MOTA) (↑): In our setup, it depends mainly on the number of ID switches.
2. ID Switches (IDSW) (↓): The absolute count of identity reassignments.
3. Identification F1 Score (IDF1) (↑): Reflects identity preservation over time.
4. Mostly Tracked (MT) (↑): The proportion of objects tracked for at least 80% in their lifetime.

Together, these metrics capture both overall tracking accuracy and long-term identity preservation. The predicted trajectories were compared with ground truths for each dataset, allowing systematic evaluation under varying velocity heterogeneity and particle densities.

## 5. Results and Discussions

**Figure 5:**
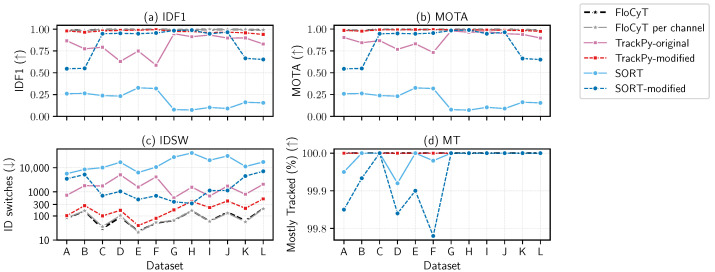
Comparison of tracking performance on artificial datasets using six algorithms: FloCyT, FloCyT per-channel, TrackPy (original and modified), and SORT (original and modified). Datsets A–F represent higher $v_{min}$, while G–L represent lower $v_{min}$ datasets. Metrics shown are (**a**) IDF1, (**b**) MOTA, (**c**) ID switches (log scale), and (**d**) Mostly Tracked (%). FloCyT and FloCyt per channel consistently achieves high IDF1 and MOTA with very few IDSW and 100% MT. TrackPy-modified was generally competitive with FloCyT, albeit with higher IDSW. TrackPy-original showed highly variable IDF1 and MOTA scores for A–F with improved performance for G–L and MT remained at 100%, but IDSW close to 1000 or more. SORT had the least favorable outcomes overall, with the lowest IDF1 and MOTA, the highest IDSW, and variable MT. SORT-modified methods outperformed SORT, particularly for datasets C–J, with competitive IDF1 and MOTA with leading algorithms but reduced performance at larger velocity ranges, exhibiting higher IDSW. Supplementary Videos S6 and S7 show tracking on synthetic dataset.

**Figure 6:**
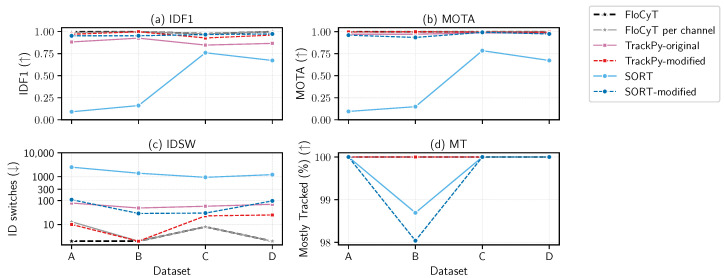
Comparison of tracking performance on four real patient datasets using six algorithms: FloCyT, FloCyT per-channel, TrackPy (original and modified), and SORT (original and modified). Metrics shown are (**a**) IDF1, (**b**) MOTA, (**c**) ID switches (log scale), and (**d**) Mostly Tracked (%). FloCyT consistently achieve near-optimal IDF1 and MOTA with very few IDSW and 100% MT, and the FloCyT per-channel variant shows similar performance with the exception of Dataset 1 (see Appendix B, Figure A2, dataset 1), which has higher IDSW due to suboptimal clustering clustering. TrackPy-modified performed competitively with FloCyT but with higher IDSW, except for dataset 2 where throughput was smallest (refer Table 1). TrackPy-original and SORT-modified show variable tracking accuracy and stability, with frequent identity switches and overall comparable performance to each other. SORT demonstrated the least favorable performance across all metrics and tested algorithms. Qualitative examples and failure cases are provided in Supplementary Videos S1 and S4.

The comparative evaluation of six tracking methodologies—FloCyT, FloCyT per-channel, TrackPy-original, TrackPy-modified, SORT, and SORT-modified—was undertaken using both synthetic and real capillary-driven microfluidic flow datasets, as demonstrated in Figure 5 and Figure 6, respectively. This assessment examines the effectiveness of each method in maintaining accurate trajectories and preserving cellular identities under high-density and heterogeneous flow conditions in capillary-driven microfluidic flow.

For artificial datasets (Table 2 and Figure 5), both FloCyT and its per channel variant demonstrated consistent performance, achieving IDF1 and MOTA scores exceeding 0.97 and 100% MT. These methods achieved the lowest identity switch counts (IDSW <= 200) among all tested trackers, even under the challenging conditions of high velocity ranges and high particle densities (datasets B, D, F, and L).

The original TrackPy framework demonstrated significant variability in IDF1 and MOTA scores. Notably, the datasets A–F, characterized by higher $v_{min}$ and $v_{max}$ (refer to Table 2), exhibited lower IDF1 and MOTA scores, especially under conditions of high cell density (higher throughput). In contrast, datasets G–L, where $v_{min}$ and velocity ranges were smaller, TrackPy achieved higher IDF1 and MOTA outcomes consistently reaching nearly 100% MT. However, the IDSW exceeded 1000 in high-density scenarios. This underscores TrackPy’s reliance on isotropic nearest-neighbor linking, which is based on small-displacement assumptions. Under high-velocity conditions with large centroid displacements, heterogeneous flow, and high cell density, these assumptions became unreliable, leading to frequent identity switches and lower accuracy. In contrast, for low displacement conditions (small $v_{min}$ and narrow velocity range) and the low density in datasets G–L, these assumptions are more applicable, resulting in improved identity preservation and tracking accuracy. The modified variant of TrackPy, benefited by our flow-aware track initialization strategy, displayed competitive performance, with IDF1 and MOTA close to those of FloCyT and its per-channel variant. However, the IDSW values for this enhanced approach remained consistently higher than those of FloCyT, indicating ongoing challenges in identity preservation due to isotropic nearest-neighbor linking.

SORT produced the least favorable tracking outcomes overall, characterized by lowest IDF1 and MOTA and the highest IDSW values (several 1000 s) among all tracking algorithms across all the datasets. The SORT-modified variant showed improvement compared to the baseline SORT, and in several datasets (C–J), even outperformed the original TrackPy in terms of IDF1 and IDSW counts. Notably, the IDF1 values for the SORT-modified variant remained very consistent compared to the baseline SORT for these datasets, underscoring the benefit of our track initialization method. Nevertheless, the consistently high IDSW values highlighted the limitations of IoU-based association strategies in heterogeneous flow environments and high speed tracking such as capillary-driven microfluidics, often resulting in insufficient overlap between predicted and detected bounding boxes. Beyond these experimental observations, such limitations also carry implications for diagnostic applications. In particular, when cell deformation metrics are clinically relevant and deformation is induced for diagnostic purposes, the IoU criterion is poorly suited, as it does not account for shape variation in deformed cells and detected bounding boxes may fail to sufficiently overlap with predicted.

**Figure A1:**
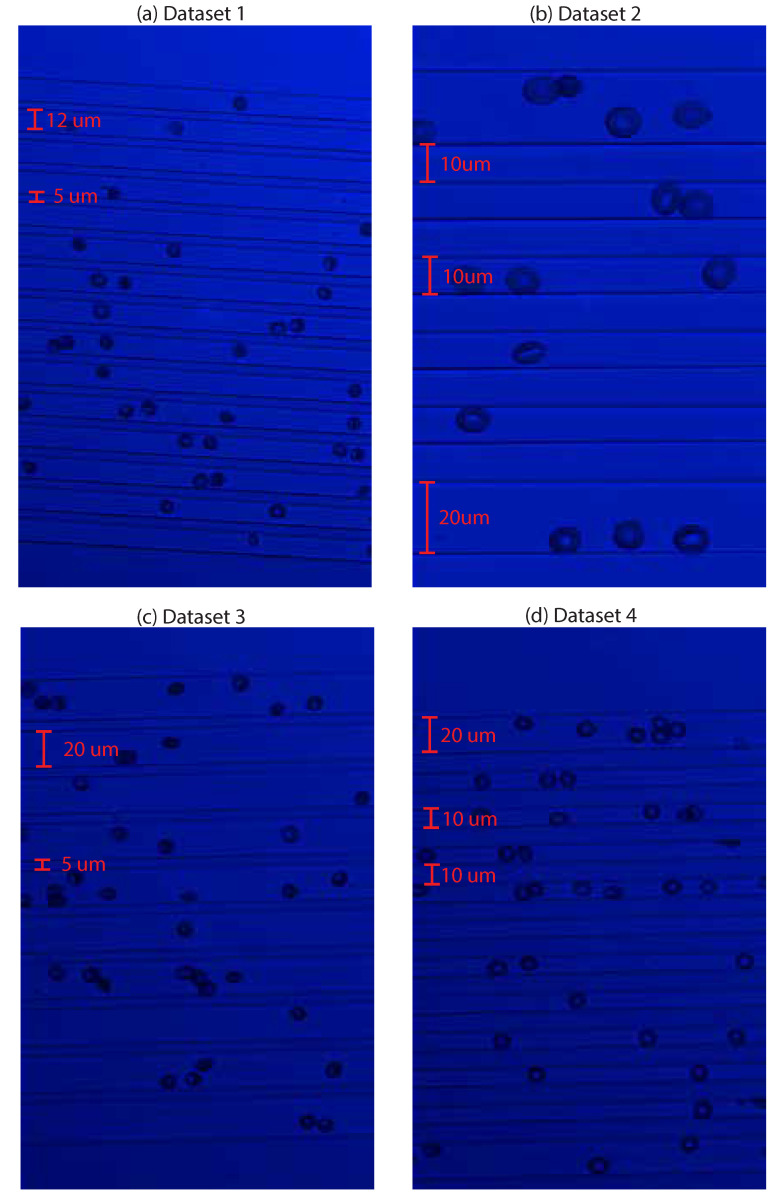
Dimensions of the channels at the FOV for the real datasets presented in Table 1. Dataset 1, acquired at 20× magnification, contains 15 channels. All channels are 12 µm wide and separated by 5 µm. Dataset 2, acquired at 40× magnification, contains 6 channels. The outer channels are 20 µm wide, while the central channels are 10 µm wide, separated by 10 µm. Dataset 3, acquired at 20× magnification, features 10 channels. All channels are 20 µm wide and separated by 5 µm. Dataset 4, acquired at 20× magnification, has 12 channels. The outer channels are 20 µm wide, and the central channels are 10 µm wide, separated by 10 µm.

**Figure A2:**
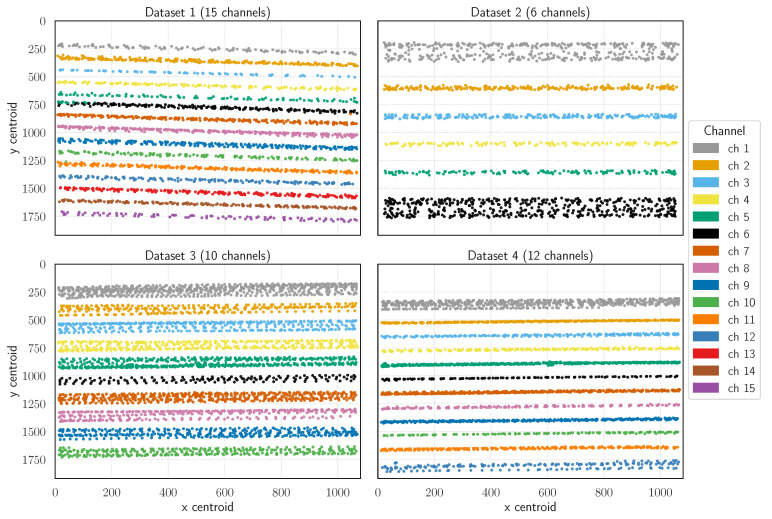
K-Means clustering of the centroids in a channel-wise fashion for real datasets, where the number of clusters corresponds to the number of parallel channels (see Table 1 for details) and width of the data in Y centroid for each channel also represents width of the microfluidic channel. Dataset 1, acquired at 20× magnification, has a slightly tilted orientation, as reflected by slanted centroid arrangements along the *y*-axis; centroid mixing is observed between channels 5 and 7 as well as channels 10 and 11 during clustering. Dataset 2, acquired at 40× magnification, contains six horizontally aligned channels that are well clustered. Dataset 3, acquired at 20× magnification, features ten slightly wider channels, leading to broader centroid distributions; it is slightly tilted but well clustered. Dataset 4, acquired at 20× magnification, has twelve horizontally aligned channels that are well clustered.

In the analysis of the real dataset (Table 1 and Figure 6), all tracking algorithms demonstrated performance trends analogous to those observed in the artificial dataset. In particular, FloCyT consistently achieved high IDF1 and MOTA values while exhibiting a reduced IDSW and 100% MT. Representative qualitative results and typical failure cases for baseline trackers are shown in Supplementary Videos S1–S5. An important exception was observed with the per-channel variant of FloCyT, which uses K-Means clustering for channel-based data association; this variant showed a decline in performance under conditions of suboptimal clustering. Such degradation was particularly pronounced in the real Dataset 1, where tilting during clip alignment led to mis-clustering (refer to Appendix A and Appendix B, Figure A1 and Figure A2, dataset 1, and Supplementary Video S5), consequently resulting cross-channel data association and higher IDSW. In the second dataset, which had the lowest throughput and well-separated centroid from higher magnification (23 cells/s; see Table 1 and Appendix A, Figure A1), the TrackPy-modified variant achieved an IDSW value that was the same as that of FloCyT. This outcome suggests that the TrackPy-modified isotropic association strategy is more effective under low-throughput conditions (lower cell density in a single frame). Nevertheless, despite these isolated instances, FloCyT overall outperformed its competitors, achieving higher IDF1 and MOTA values along with a greater proportion of tracked cells while maintaining fewer IDSWs.

## 6. Conclusions

In comparative evaluation of artificial and real capillary-driven microfluidic flow datasets, FloCyT consistently outperforms existing tracking methodologies. The framework achieves high scores in the IDF1 and MOTA metrics, with all centroids characterised as mostly tracked, and significantly reduces the occurrence of identity switches. These outcomes highlight FloCyT’s robustness in challenging scenarios characterised by complex flow dynamics, high particle densities, and heterogeneous trajectories inherent to capillary-driven systems. Although the modified TrackPy approach demonstrated improved competitiveness, particularly under low-throughput conditions, and the SORT-modified method benefited from improved initialisation strategies, both alternatives were limited by higher IDSWs.

This advancement enhances the quantitative optical sensing capability of capillary-driven microfluidic platforms, enabling accurate identity-resolved cell tracking in point-of-care diagnostics and clinical research, where high-throughput, single-cell analysis is required at a low cost and with simpler instrumentation. Future work will focus on integrating FloCyT with embedded optical sensing modules to achieve real-time, automated diagnostics at the point-of-care.

## Figures and Tables

**Table 1:** Details of the annotated real patient data used for tracking algorithm evaluation. The mean velocity is measured in pixels/frame.

Dataset	Frames	Total Cells	Mean Velocity	Throughput (Cells/s)
1	90	172	54	57
2	192	153	31	23
3	95	151	28	47
4	90	145	86	48

**Table 2:** Details of the simulated datasets. Velocities are measured in pixels/frame, and total cells indicate the total number of initialized cells for the entire simulation.

Dataset	$v_{\min}$	$v_{\max}$	Total Cells	Throughput (Cells/s)
A	100	500	2000	40
B	100	500	3000	60
C	200	300	3000	60
D	200	300	5000	100
E	300	400	3000	60
F	300	400	5000	100
G	50	100	2000	40
H	50	100	3000	60
I	50	150	2000	40
J	50	150	3000	60
K	50	300	2000	40
L	50	300	3000	60

## Data Availability

The data supporting the findings of this study are available from the corresponding author upon reasonable request.

## Supplementary Materials

The following supporting information can be downloaded at https://www.mdpi.com/article/10.3390/s25227040/s1, Supplementary File S1; Supplementary Videos S1–S7 provide side-by-side comparisons of different tracking algorithms. Videos S1–S4 show tracking results on real datasets recorded under various channel geometries. Video S1 compares Ground Truth, FloCyT, TrackPy-original, and SORT in two parallel channels, highlighting typical failure modes of TrackPy-original, while SORT frequently struggles with track linking and repeatedly reinitializes new track IDs. FloCyT addresses these errors by applying anisotropic search gating aligned with the flow direction. Video S2 demonstrates FloCyT’s consistent performance under time-varying flow velocities at higher magnification and compares it with Ground Truth, TrackPy-modified, and SORT-modified. Video S3 compares the robustness of FloCyT, TrackPy-modified, and SORT-modified in wider parallel channels. Video S4 highlights issues similar to those seen in Video S1, but focuses on the modified versions of TrackPy and SORT; TrackPy-modified exhibits fewer bouncing-back issues, though close centroid swaps persist, and SORT-modified achieves better linking performance than the original SORT, although reinitialization errors still occur. Video S5 compares Ground Truth, FloCyT, FloCyT per channel, and TrackPy-modified, showing that imperfect clustering in tilted channel geometries can cause cross-channel identity switches. Finally, Videos S6 and S7 present artificial datasets used for benchmarking. Video S6 compares Ground Truth, FloCyT, TrackPy-original, and SORT, while Video S7 compares Ground Truth, FloCyT, TrackPy-modified, and SORT-modified.

## Abbreviations

The following abbreviations are used in this manuscript:

POC	point-of-care
PMMA	polymethyl methacrylat
LAP	linear assignment problem
SORT	simple online real time
ROI	region-of-interest
IoU	intersection-over-union
RBC	red blood cell
MOTA	multiple object tracking accuracy
IDSWs	ID switches
MT	mostly tracked
FOV	field of view

## Appendix A. Dimensions of Grid Structures at FOV of Chips

Figure A1 provides the dimensions of the channels at the FOV for the real datasets presented in Table 1. Dataset 1 represents an ideal channel geometry in which all channels are evenly spaced and uniform in width, making it suitable for cytometry-style applications. Dataset 2 was designed to accommodate fewer channels at higher magnification, while Dataset 4 provides more channels at lower magnification. Both Dataset 2 and Dataset 4 include two different channel sizes, where one is twice as wide as the other, allowing evaluation of the tracking algorithm’s adaptability to varying channel widths within the same FOV. Finally, Dataset 3 features wider channels that can accommodate 2–3 cells within the same cross-section. This design enables the observation of more dynamic flow behaviors, such as cells moving side-by-side or overtaking each other.

## Appendix B. Effectiveness of the K-Means Clustering

Figure A2 illustrates the channel-wise K-Means clustering results for the four real patient datasets used for tacking assessment. The detected centroids are color-coded by cluster, showing the geometric layout and separation of parallel channels in each sample. The figure caption provides details on channel alignment, cluster quality, and magnification settings for each dataset.
