# Supplementary material for: FloCyT: A Flow-Aware Centroid Tracker for Cell Analysis in High-Speed Capillary-Driven Microfluidic Flow
Source: Sensors (Basel). 2025 Nov 18;25(22):7040. doi: 10.3390/s25227040 (PMC12656555; doi:10.3390/s25227040)
Supplement: Supplementary file 1 [file sensors-25-07040-s001.zip › File S1_FloCyT.pdf]

Supplementary Materials: FloCyT: A Flow-Aware Centroid Tracker for Cell Analysis in High-Speed Capillary-Driven Microfluidic Flow

Suraj K. Maurya [1,2,*] , Matt Stark[2], Cédric Bessire[2]

[1]Bio/CMOS Interfaces Laboratory, Ecole Polytechnique Federale de Lausanne (EPFL) , rue de la Maladiere 71, 2000, Neuchâtel, Switzerland
[2]Institute For Human Centered Engineering, Berner Fachhochschule (BFH), Quellgasse 21 ,2501 Biel, Switzerland

[*] Correspondence: suraj.maurya@bfh.ch

1 Supplementary videos for side-by-side comparison of the different trackers.

All the videos are encoded at 30 fps, except for S1 and S4, they encoded at 7fps

1.1 Supplementary Video S1 - Real Dataset 1

Side-by-side comparison of Ground Truth, FloCyT, TrackPy, and SORT. We compare 2 parallel channels and highlight typical failure modes of baseline trackers. TrackPy frequently produces backward trajectory jumps when cells enter or exit the field of view (red boxes), due to isotropic gating and memory-based linking. The modified TrackPy shows this behaviour even in the modified version, where we used our initialisation strategy (also visible in S4). FloCyT reduces backward linking by applying anisotropic search gating aligned with the flow direction. This allows a wider search in the direction of flow and a smaller search range in the backward directions. TrackPy also exhibits identity swaps at higher particle densities (green boxes). SORT struggles with large inter-frame displacements, leading to frequent premature initialisation of new tracks; the modified SORT improves performance but still shows intermittent new initialisation of tracks.

1.2 Supplementary Video S2 - Real Dataset 2 (Higher Magnification And Wider Outer Channels).

Comparison of FloCyT, modified TrackPy, and modified SORT. Flow velocity varies over time, with FloCyT maintaining consistent identity assignment under changing displacement magnitudes.

1.3 Supplementary Video S3 - Real Dataset 3 (Wider Channel)

Comparison of FloCyT, modified TrackPy, and modified SORT. FloCyT preserves identity in wider channels.

1.4 Supplementary Video S4 - Real Dataset 4 (Wider Outer Channels)

Comparison of FloCyT, modified TrackPy, and modified SORT. Highlight backwards-linking issues for modified TrackPy, like the S1.

1.5 Supplementary Video S5 - Real Dataset 1.

Comparison of ground truth FloCyT, FloCyT-KMeans, and modified TrackPy. S5 illustrates cross-channel identity swaps in the KMeans-based variant, resulting from imperfect clustering in the tilted channel geometry. This led to cross-channel identity switches.

1.6 Supplementary Video S6 and S7 - Artificial Dataset i

Snippet to show synthetic data. S6 shows the comparison of ground truth, FloCyT, TrackPy and SORT. S7 shows a comparison of ground truth, FloCyT, FloCyT, modified TrackPy and modified SORT. The typical error of the bouncing back of tracks is observed in the original TrackPy version (multiple occasions, for example, between 326 and 334), as well as a close centroid switch (Track ID 349 switches multiple times). Whereas in the S7 version, a centroid switch is observed (Track ID 350 switches multiple times). SORT shows frequent new ID creation, and its modified version shows less frequent new ID creation.
